# Secretory Production of Plant Heme-Containing Globins by Recombinant Yeast via Precision Fermentation

**DOI:** 10.3390/foods14081422

**Published:** 2025-04-20

**Authors:** Ha-Neul Bae, Geun-Hyung Kim, Seung-Oh Seo

**Affiliations:** 1 Department of Food Science and Biotechnology, Seoul National University of Science and Technology, Seoul 01811, Republic of Korea; qogksmf0508@seoultech.ac.kr (H.-N.B.); gh.kim@seoultech.ac.kr (G.-H.K.); 2 Research Institute of Food and Biotechnology, Seoul National University of Science and Technology, Seoul 01811, Republic of Korea

**Keywords:** *Saccharomyces cerevisiae*, *Komagataella phaffii*, plant leghemoglobin, secretion, precision fermentation

## Abstract

Leghemoglobin (LegHb) is a plant-derived heme-containing globin found in the root nodules of legumes like soybean that can be used as a food additive for red color and meaty flavor as a plant-based meat alternative. However, conventional extraction methods face challenges of low yield and high costs. To address this issue, precision fermentation with recombinant microorganisms has been applied for the sustainable large-scale production of plant leghemoglobins. This study attempted the production of plant legHbs using recombinant yeast strains, *Saccharomyces cerevisiae* and *Komagatella phaffii*. The plant legHb genes were identified from the genome of legumes such as soybean, chickpea, mung bean and overexpressed in yeast via extracellular secretion by the signal peptide and inducible promoters. Subsequently, hemin as a heme provider was added to the fermentation, resulting in increased levels of plant legHbs. In *S. cerevisiae*, gmaLegHb expression reached up to 398.1 mg/L, while in *K. phaffii*, gmaLegHb showed the highest production level, reaching up to 1652.7 mg/L. The secretory production of plant legHbs was further enhanced by replacing the signal peptide in the recombinant yeast. The secreted plant legHbs were purified by His-Tag from a culture supernatant or concentrated via precipitation using ammonium sulfate. These results suggest that the production of plant legHbs is significantly influenced by hemin and signal peptide. This study successfully demonstrates the production of the various plant legHbs other than soy legHb that can be used as natural colors and flavors for plant-based meat alternatives.

## 1. Introduction

Meat is crucial food for human development and maintenance, providing essential amino acids, fatty acids, and vitamins [1]. According to the Food and Agriculture Organization of the United Nations (FAO), the global increase in population and other factors are leading to a rise in meat consumption, which is consequently causing issues related to supply shortages and environmental pollution [2]. Environmental concerns associated with farming include the pollution of various resources such as land and water, as well as the greenhouse gases emitted by animals [3]. To solve these problems, there is increasing interest in alternative meat products and sustainable food ingredients [4].

The development of alternative meats can be broadly categorized into cultured meat, edible insects, and plant-based meat [5]. Among these options, development is particularly active for plant-based meat due to increasing interest in vegetarianism [6]. Plant-based meat has a decent protein content that is comparable to traditional meats and has a high proportion of essential amino acids from soybeans [7,8]. Additionally, plant-based meat is notable as an alternative food due to its low content of fats and saturated fatty acids [9]. However, plant-based meat products need to improve sensory quality levels, such as color and taste, compared to animal meats [10]. Recently, various research and development have been conducted to address these issues by incorporating various food additives, mimicking the taste and color of traditional meats as closely as possible [11].

Heme-containing proteins such as soy leghemoglobin (LegHb) are used as food additives for plant-based meats to provide the meaty color and blood flavor of meat [5,12]. LegHb is a small monomeric protein, approximately 15–16 kDa in size, found in the root nodules of legumes such as soybeans [13,14]. This protein consists of a globin subunit bound to heme that is similar to myoglobin in animals [15]. An extraction method for legHb production from soybean root nodules is inefficient for large-scale production [16]. To address this, a recombinant legHb has been produced through microbial fermentation processes using various microorganisms [17,18,19,20]. While most studies have focused on soybean-derived legHbs, the expression and activity of legHb variants from other plants remain underexplored, despite their potential advantages such as allergenicity reduction [21,22].

For the recombinant legHb production, heme is an essential factor for increasing the expression levels and activities of legHbs. In the recombinant *Komagatella phaffii*, the addition of hemin, which is a ferric chloride heme, was helpful in increasing the activity of legHb, while the addition of aminolevulinic acid (ALA) enhanced the activity and protein concentration of legHb [23]. Currently, the recombinant *K. phaffii* shows the highest yield of legHb [24]. *K. phaffii* is a valuable host for recombinant protein production due to its capacity to achieve high cell densities and its robust protein expression capabilities [25]. *Saccharomyces cerevisiae* is a yeast strain that has been used for food production for a long time and is recognized as a potential platform host for protein production [24]. Both yeast strains are known as GRAS (generally recognized as safe), which can be used as safe industrial hosts for the production of food ingredients [26]. The yeast strains perform post-translational modifications, allow reactions to be segregated within subcellular organelles, and are more resistant to viral infections [27]. In addition, compared to bacterial hosts, yeast provides a eukaryotic environment that allows for complex post-translational modifications such as glycosylation and disulfide bond formation, which are often essential for the correct folding and function of plant proteins [28]. This is a major advantage of yeast expression systems for the production of plant proteins.

This study attempts to express various plant-based legHbs derived from leguminous plants in both *S. cerevisiae* and *K. phaffii*. Novel plant legHbs other than soy legHb are selected from the genome of leguminous plants, including mung bean, chickpea, and cowpea, based on structural similarity with the soy legHb. To express and secrete the selected plant legHbs in yeast, inducible promoters and signal peptides are employed. Additionally, several approaches to enhance the production levels of plant legHbs by recombinant yeast are performed. The legHbs produced in this study are expected to be utilized in various industrial applications for the development of food colorants, food flavors, pharmaceuticals, and serum-free culture media for cultured meat.

## 2. Material and Methods

### 2.1. Strains and Plasmids

All strains and plasmids used in this study are listed in Table 1. Plasmid pESC-URA and pPICzαA were obtained from Invitrogen (Carlsbad, CA, USA). The gene sequences of *Glycinin max* (soybean) LegHb (gmaLegHb, GenBank accession number: NP_001235248.2), *Cicer arietinum* (chickpea) LegHb (carLegHb, GenBank accession number: LOC101496383), *Vigna radiata* var. *radiata* (mung bean) LegHb (vraLegHb, GenBank accession number: LOC106771851), *Trifolium pratense* (red clover) LegHb (tprLegHb, GenBank accession number: PNY06089.1), *Vigna unguiculata* (cowpea) LegHb (vunLegHb, GenBank accession number: AAB65769.1) were obtained from NCBI and then codon-optimized and synthesized by Integrated DNA Technologies (Coralville, IA, USA).

The pESC-URA plasmid was used for the construction of the plant Hbs expression plasmid in *S. cerevisiae* D452-2. For all pESC-URA backbone constructions, the BamHI restriction enzyme (Takara Bio Inc., Kusatsu, Japan) was used, and PrimeSTAR, along with a thermocycler, was utilized for insert constructions. The pPICZαA plasmid was utilized for the construction of the plant legHbs expression plasmid in *K. phaffii* X-33. All pPICZαA backbone and insert constructions used PrimeSTAR (Takara Bio Inc., Kusatsu, Japan) and a thermocycler (Bio-Rad, Hercules, CA, USA). All DNA fragments were assembled using Gibson Assembly Master Mix (New England Biolabs Inc., Ipswich, MA, USA) to construct recombinant plasmid.

The α-mating factor (MFα) signal peptide was replaced with a different signal peptide in pPICZαA and pESC-URA expression plasmids. The MFα signal peptide was from pPICZαA plasmid. The signal peptides of serum albumin from *Homo sapiens* and α-amylase from *Aspergillus niger* were synthesized by Integrated DNA Technologies (Coralville, IA, USA).

### 2.2. Media and Culture Conditions

*Escherichia coli* TOP10 was used for cloning and propagation. *E. coli* TOP10 was cultivated at 37 °C and 200 rpm in Luria-Bertani (LB) medium (5 g/L yeast extract, 10 g/L tryptone, and 10 g/L NaCl) with ampicillin (100 ug/mL) or Zeocin (50 ug/mL).

*S. cerevisiae* D452-2 and *K. phaffii* X-33 were used as the plant legHb gene expression host. *K. phaffii* X-33 was cultivated at 30 °C and 200 rpm in Buffered Glycerol-complex (BMGY) medium (10 g/L yeast extract, 20 g/L peptone, 13.4 g/L yeast nitrogen base, 100 mM potassium phosphate pH 6.0, 0.4 mg/L biotin, and 10 g/L glycerol and Buffered Methanol-complex (BMMY) medium (10 g/L yeast extract, 20 g/L peptone, 13.4 g/L yeast nitrogen base, 100 mM potassium phosphate pH 6.0, 0.4 mg/L biotin, and 5 g/L methanol). The cells were initially cultured in BMGY medium for 24 h. After 24 h, the cells were harvested via centrifugation at 3663 g for 30 min. The collected cells were resuspended and inoculated into BMMY medium at the initial OD_600_ = 4.0 and cultivated for 72 h. To maintain induction conditions, methanol was added to the culture at a final concentration of 0.5% every 24 h.

*S. cerevisiae* D452-2 was cultivated at 30 °C and 200 rpm in synthetic dropout (SC) medium (6.7 g/L yeast nitrogen base without amino acids, 0.6 g/L complete supplement mixture without histidine, leucine, tryptophan, uracil, 5 g/L ammonium sulfate, and 50 mg/L histidine, 50 mg/L leucine, 50 mg/L tryptophan) with 20 g/L glucose and yeast extract peptone (YP) medium (10 g/L yeast extract, 20 g/L peptone) with 20 g/L galactose. Yeast cells were initially cultured in SC medium lacking uracil for 24 h. Following this incubation period, the cells were harvested via centrifugation. The collected cells were inoculated in a YP medium containing galactose at the initial OD_600_ = 4.0. The culture was then incubated for 72 h to induce the legHb protein expression.

### 2.3. Protein Purification and Quantification

To purify the recombinant plant legHbs using His-Tag, the fermentation supernatants were harvested via centrifugation and purified using a Ni–NTA agarose (Quiagen, Hilden, Germany). Then, 1 mL of Ni-NTA agarose resin was loaded into a gravity column (Bio-Rad, Hercules, CA, USA). Then, 50 mL of supernatants was transferred into the gravity column and incubated for 1 h. Afterwards, elution was performed using a 250 mM imidazole buffer. The purified fraction was mixed with sample buffer and heated at 95 °C. The purified fraction was analyzed with 15% (*w*/*v*) SDS-PAGE gel. The plant legHbs concentration was determined using the Bradford Protein Assay kit (Takara Bio Inc., Kusatsu, Japan). The mean density of the target legHb protein band was analyzed using Image-J software (version 1.54K; National Institutes of Health, Bethesda, MD, USA).

### 2.4. Peroxidase Activity Assay for Plant Hemoglobin

Purified supernatant was mixed with TMB Substrate Solution from Peroxichrom Excel™ (Genomine, Pohang, Korea) and incubated at 37 °C in the dark for 5 min. Subsequently, the reaction was stopped by adding 2 M H_2_SO_4_ [19]. Absorbance detected by Synergy HTX multi-mode reader (BioTek, Winooski, VT, USA) at 450 nm was converted to the concentration of TMB-derived oxidation products. The calculation was performed using the Beer–Lambert Law with an extinction coefficient of 59,000 M^−1^ 1 cm^−1^. One unit of the peroxidase activity was defined as the amount of enzyme that catalyzed the 1 μmol TMB-derived oxidation products per minute.

### 2.5. Precipitation of Recombinant Proteins by Ammonium Sulfate

Induced yeast cell culture for 72 h was centrifuged at 3663× *g* for 30 min. The supernatant was precipitated with ammonium sulfate for 50% saturation [29]. Ammonium sulfate was added slowly to the supernatant at 4 °C with stirring. The ammonium sulfate mixture was centrifuged at 3663× *g* for 30 min at 4 °C. After discarding the supernatant, the precipitate was re-suspended in 1 mL of 100 mM potassium phosphate buffer and then dialyzed at 4 °C for 24 h. The precipitates in the buffer were mixed with a sample buffer and heated at 95 °C. The precipitate sample was analyzed with 15% (*w*/*v*) SDS-PAGE gel.

### 2.6. Statistical Analysis

At least three replicates were performed for all experiments. Results were expressed as the average with standard errors. All statistical analyses were conducted with SAS 9.4 from SAS Institute Inc. (Cary, NC, USA). Comparisons between two groups were performed using Student’s *t*-test, and comparisons among three or more groups were conducted using one-way analysis of variance (ANOVA). A *p*-value < 0.05 was considered statistically significant.

## 3. Results

### 3.1. Secretory Production of Plant Leghemoglobin in Recombinant Yeast

Genes for plant legHbs from various leguminous plants were identified and expressed. The five selected legHb genes (gmaLegHb, carLegHb, vraLegHb, tprLegHb, and vunLegHb) with the MFα signal peptide were inserted into the pESC-URA and pPICZαA plasmids (Figure S1) and transformed into *S. cerevisiae* and *K. phaffii*, respectively.

**Figure 1:**
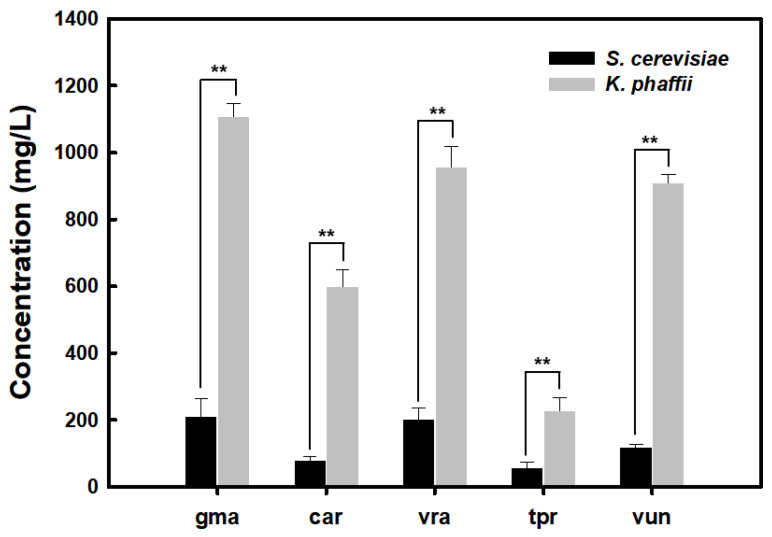
Quantification of plant leghemoglobins secreted by *S. cerevisiae* and *K. phaffii* with MFα signal peptide. Statistical significance was determined by Student’s *t*-test. ** *p* < 0.05.

**Figure 2:**
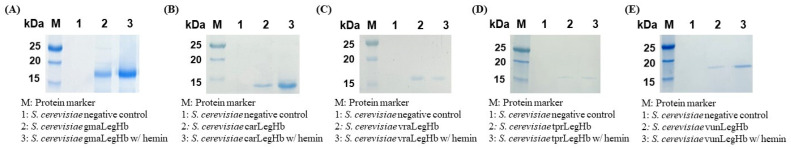
SDS-PAGE analysis of purified plant leghemoglobins secreted by recombinant *S. cerevisiae* with hemin supplementation. (**A**) gmaLegHb, (**B**) carLegHb, (**C**) vraLegHb, (**D**) tprLegHb, (**E**) vunLegHb.

For the legHb expression in *S. cerevisiae*, galactose was used to induce protein expression in the selected colonies of transformants. Similarly, the plant legHb expression plasmids, linearized by Sacl and transformed into *K. phaffii*, and selected transformants on YPD agar plates containing zeocin were induced for expression using methanol as an inducer. As a result, all plant legHb were successfully expressed by inducible promoters and secreted by the MFα signal peptide in the recombinant *S. cerevisiae* and *K. phaffii* (Figure 1). However, concentrations of the plant legHb proteins produced by the recombinant *S. cerevisiae* and *K. phaffii* showed significant differences among the genes and host strains. The concentrations of all plant legHbs were significantly higher in *K. phaffii* compared to that in *S. cerevisiae*. This result suggested that *K. phaffii* is a suitable host strain for expressing recombinant plant legHbs. The higher expression of legHbs in *K. phaffii* may result from the strain’s high capacity for protein expression and its rapid growth rate [25]. Also, the expression levels of legHb proteins varied based on the source of genes. In both *S. cerevisiae* and *K. phaffii*, the gmaLegHb protein showed the highest production level, while the tprLegHb protein showed the lowest production level. This was also confirmed by the SDS-PAGE analysis (Figure 2). These findings indicate that the expression levels of legHbs may be influenced by both the host strain and the protein source. Therefore, this study aimed to overexpress various plant legHbs in different yeast strains.

### 3.2. Effect of Hemin Supplementation on Plant Leghemoglobin Production

The production of LegHb may be limited due to a deficiency in the heme cofactor and saturation of the secretion [23]. Previously, supplementation of 10 uM hemin to the medium was effective in enhancing the secretion efficiency [30]. To enhance the expression level of legHb as a holoprotein bound to heme, hemin was supplemented in the fermentation medium. *S. cerevisiae,* grown in the SC medium lacking uracil, with glucose were transferred to YP galactose medium with and without hemin. Similarly, *K. phaffii* cells grown in the BMGY medium were inoculated into the BMMY medium containing methanol with and without hemin supplementation. To confirm the functionality of plant legHbs secreted by recombinant yeast, a peroxidase activity assay was performed since the heme in the medium harbors intrinsic peroxidase activity [23].

**Figure 3:**
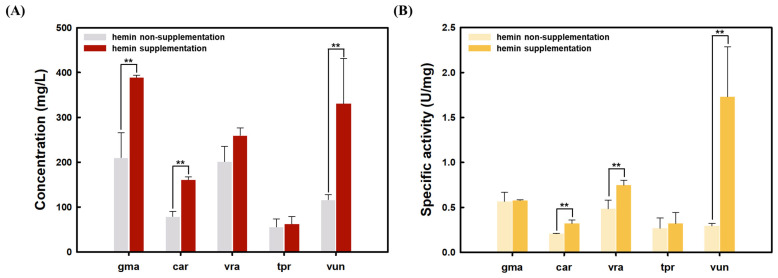
Effect of hemin supplementation on plant leghemoglobin secretory productions in *S. cerevisiae.* (**A**) Quantification of plant leghemoglobin protein concentration in *S. cerevisiae* with hemin supplementation (**B**) Specific activity of plant leghemoglobins in *S. cerevisiae* with hemin supplementation. Statistical significance was determined by Student’s *t*-test. ** *p* < 0.05.

As a result, the band thickness of all the purified plant legHb proteins produced by recombinant *S. cerevisiae* in the SDS-PAGE analysis increased with 10 uM hemin supplementations compared to that without hemin (Figure 2). Particularly, gmaLegHb, carLegHb, and vunLegHb significantly increased protein concentrations when the hemin was added to the medium (Figure 3). The peroxidase activities of vraLegHb, vunLegHb, and carLegHb produced by *S. cerevisiae* significantly increased upon hemin supplementation (Figure 3).

**Figure 4:**
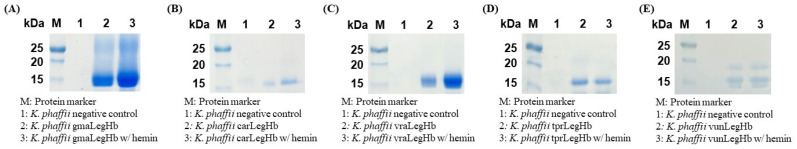
SDS-PAGE analysis of purified plant leghemoglobins secreted by recombinant *K. phaffii* with hemin supplementation. (**A**) gmaLegHb, (**B**) carLegHb, (**C**) vraLegHb, (**D**) tprLegHb, (**E**) vunLegHb.

**Figure 5:**
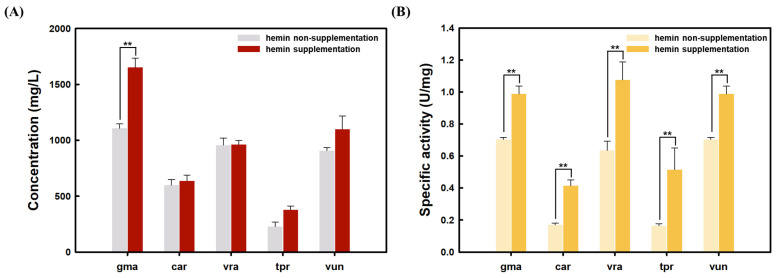
Effect of hemin supplementation on plant leghemoglobin secretory productions in *K. phaffii.* (**A**) Quantification of plant leghemoglobin protein concentration in *K. phaffii* with hemin supplementation. (**B**) Specific activity of plant legHbs in *K. phaffii* with hemin supplementation. Statistical significance was determined by Student’s *t*-test. ** *p* < 0.05.

In *K. phaffii*, all plant legHbs showed increased levels of protein concentration with the hemin supplementation (Figure 4). gmaLegHb showed the most significant increase in concentration with hemin in *K. phaffii* (Figure 5). Moreover, all of the plant legHbs produced by recombinant *K. phaffii* exhibited increased peroxidase activities with the addition of hemin (Figure 5). These results suggest that hemin supplementation can help to increase the plant legHb expression as well as its activity in the recombinant yeast. This observation is consistent with previous reports [30,31]. Several studies have demonstrated that in yeast, the addition of hemin significantly enhances the peroxidase activity of recombinant heme-containing apoproteins without altering their expression levels. These studies concluded that hemin facilitates the conversion of secreted apoproteins into catalytically active holoproteins, thereby increasing the overall specific activity [30,31].

### 3.3. Precipitation of Plant Leghemoglobin Using Ammonium Sulfate

To enhance the economic feasibility of legHb production from recombinant yeast, ammonium sulfate can be used for precipitation, allowing for an efficient initial protein concentration. The secreted legHb protein in the medium can be easily harvested using the precipitation method, reducing processing costs while maintaining protein integrity with a scalable purification process. To test whether the plant legHb proteins secreted by the recombinant yeast can be harvested via precipitation, we performed salt precipitation using ammonium sulfate. After that, the plant legHb precipitation was confirmed by SDS-PAGE analysis (Figure S2). Distinct bands of the precipitated plant legHb proteins were observed in both *S. cerevisiae* and *K. phaffii*. However, the band appeared smeared vertically, indicating possible protein aggregation, degradation, or improper sample preparation. These results suggest that ammonium sulfate precipitation can be used for the preparation of legHb protein concentrates with low purity. When a high purity of legHb protein is necessary, affinity chromatography using His-Tag appears to be a more effective method, likely providing higher purity and yield.

### 3.4. Replacement of Signal Peptide for Enhanced Secretion of Plant Leghemoglobin

**Figure 6:**
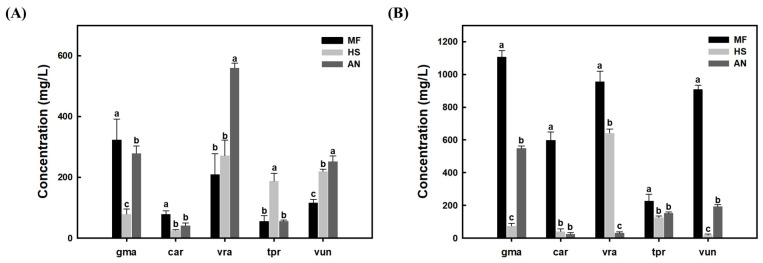
Comparison of plant hemoglobin secretory productions in recombinant yeast with different signal peptides. (**A**) Leghemoglobin concentrations produced by the recombinant *S. cerevisiae* and (**B**) by the recombinant *K. phaffii* strains. Different letters (a, b, c) indicate significant differences among groups based on one-way ANOVA followed by Duncan’s multiple range test (*p* < 0.05).

The efficiency of secretory signal peptides also plays a crucial role in influencing the expression of heterologous proteins [32]. The MFα signal peptide from *S. cerevisiae,* which is the most commonly used signal peptide [33], was initially used for the secretory production of plant legHbs in *S. cerevisiae* and *K. phaffii* in this study. To test other signal peptides for the enhanced secretion of the plant legHb proteins in yeast, the MFα signal peptide sequence was replaced with the serum albumin signal peptide sequence from *Homo sapiens* (HS) and α-amylase signal peptide sequence from *Aspergillus niger* (AN) in the expression plasmids. The fermentation results showed differences in the expression level of each plant legHb in *S. cerevisiae* after the signal peptide replacement (Figure 6A). The highest protein level was observed in the vraLegHb expression with AN signal peptide. According to the SDS-PAGE analysis, there was the appearance of several additional protein bands other than vraLegHb (Figure S3). Thus, the vraLegHb with the MFα signal peptide was still the most effective signal peptide for secretion in *S. cerevisiae*. Also, the gmaLegHb and carLegHb with the MFα signal peptide showed the best results among the three signal peptides tested in *S. cerevisiae* (Figure 6A). However, tprLegHb with the HS signal peptide significantly increased the secretion level. Also, vunLegHb showed increased secretion levels after the replacement with the HS signal peptide and the AN signal peptide. Overall, when the signal peptide was replaced, some legHb proteins showed increased secretion, while others did not. This suggests that specific signal peptides may enhance secretion efficiency depending on the protein. Similar observations have been reported in previous studies, showing that the compatibility between the signal peptide and the protein significantly affects secretion results [34,35].

In *K. phaffii*, all plant legHb proteins showed the highest secretion level with the MFα signal peptide, suggesting that the HS and AN signal peptides were not helpful in enhancing the secretion levels (Figure 6B). These results were also confirmed by SDS-PAGE analysis (Figure S4). The secretion efficiency of signal peptides varies for the same protein depending on the host strain. It is noted that a single signal peptide cannot guarantee the secretion of all proteins in an expression host [19]. Based on these results, we can conclude that selecting the optimal signal peptide for target proteins and host strains is crucial for achieving efficient secretion.

## 4. Discussion

Heme-containing globin protein has great potential as a key food additive that provides a meaty flavor and red color to plant-based meat. LegHb, a heme-containing protein derived from soybean, was approved by the FDA in 2019 as an additive for vegetarian meats [36]. LegHb can be produced via extraction from the root nodules of leguminous plants like soybeans. However, this method has limitations in producing legHb on a large scale with high yield. Thus, this study focused on the microbial production of plant LegHb using recombinant yeast strains via precision fermentation. In this study, novel plant Hbs with structures similar to soy LegHb were first expressed with an inducible promoter and the MFα signal peptide in the GRAS yeast *S. cerevisiae* and *K. phaffii*. The donors of the plant legHb genes were legume plants that have a long history of human consumption, including soybean, chickpea, mung bean, red clover, and cowpea.

Secretory production can simplify the downstream purification steps by harvesting the fermentation medium harboring target proteins without the cells [37]. When the same plant Hb was expressed in both strains, the expression level in *K. phaffii* was higher than in *S. cerevisiae*. This suggests the *K. phaffii* strain is suitable for the high production of plant legHb. However, methanol was used as an inducer for *K. phaffii.* Methanol induction may not be suitable for food applications. In *S. cerevisiae*, galactose was used as an inducer that is much safer than methanol.

For the downstream process, we tested the feasibility of precipitation methods for purifying plant legHb using ammonium sulfate without affinity chromatography, making it suitable for large-scale protein production. Ammonium sulfate precipitation offers significant advantages for protein purification. This method is cost-effective and simple, allowing for the efficient processing of large-scale purification with minimal equipment requirements [38,39]. This study confirmed that the precipitation method using ammonium sulfate can be feasible if the precipitation process is further optimized for stable protein preparation.

Heme is an essential factor that influences heme-containing proteins such as legHbs [40]. This study confirmed that the addition of hemin to the culture medium increased the protein expression levels and activities of some plant legHbs in the recombinant yeast. Secretion is an efficient method for protein production as it provides straightforward and simple purification. To increase the secretion levels of legHb proteins, several signal peptides from eukaryotes, including *S. cerevisiae*, *H. sapiens*, and *A. niger,* were evaluated in *S. cerevisiae* and *K. phaffii*. These observations are in line with previous studies reporting that the interplay between the signal peptide, the host, and the structure of the recombinant protein collectively influences secretion levels [41,42,43]. Further exploration using a broader library of signal peptides and direct biochemical validation could help refine the optimization strategy for efficient LegHb secretion.

As a result, we confirmed that one signal peptide cannot efficiently secrete all heterologous proteins in various hosts. A proper signal peptide is a key factor that affects the efficiency of protein secretion.

Overall, this study successfully demonstrated the secretory production of various plant legHbs in *S. cerevisiae* and *K. phaffii*. This investigation represents the first secretory production of novel plant legHbs other than soy legHb, a well-known food additive used by alternative meat companies. Signal peptide engineering to enhance secretion levels, along with metabolic engineering to boost intracellular heme content, can be further applied to develop microbial cell factories for the cost-effective large-scale production of plant legHbs.

## 5. Conclusions

In this study, plant legHbs were successfully expressed and secreted by the yeasts *S. cerevisiae* and *K. phaffii* with inducible promoter and signal peptides. For both strains, the addition of hemin to the fermentation medium resulted in increased protein concentrations and activities of plant legHbs compared to in the absence of hemin. Several signal peptides were employed for enhanced protein secretion in yeast, resulting in increased secretion levels of plant legHbs. This study demonstrates the power of precision fermentation for the sustainable production of food ingredients such as colors and flavors using recombinant microorganisms. Considering the increasing global population and the instability of the food supply caused by rapid climate change, the development of new technologies to improve food production while reducing environmental impacts is of critical importance. The results of this study suggest the potential of precision fermentation for the more efficient and scalable production of food ingredients, contributing to the development of environmentally friendly and sustainable alternative food sources, with significant implications for the food industry.

## Figures and Tables

**Table 1:** Strains and plasmids used in this study.

Name	Description	Reference
*S. cerevisiae* D452-2	*MATα*, *leu2*, *his3*, *ura3*, and *can1*	[24]
pESC-MFα-gmaHb	D452-2 URA3, P_GAL1_-MFα- *Glycinin max* Hb	This study
pESC-MFα-carHb	D452-2 URA3, P_GAL1_-MFα- *Cicer arietinum* Hb	This study
pESC-MFα-vraHb	D452-2 URA3, P_GAL1_-MFα- *Vigna radiata* var. *radiata* Hb	This study
pESC-MFα-tprHb	D452-2 URA3, P_GAL1_-MFα- *Trifolium pratense* Hb	This study
pESC-MFα-vunHb	D452-2 URA3, P_GAL1_-MFα- *Vigna unguiculata* Hb	This study
pESC-HS-gmaHb	D452-2 URA3, P_GAL1_-HS- *G. max* Hb	This study
pESC-HS-carHb	D452-2 URA3, P_GAL1_-HS- *C. arietinum* Hb	This study
pESC-HS-vraHb	D452-2 URA3, P_GAL1_-HS- *V. radiata* var. *radiata* Hb	This study
pESC-HS-tprHb	D452-2 URA3, P_GAL1_-HS- *T. pratense* Hb	This study
pESC-HS-vunHb	D452-2 URA3, P_GAL1_-HS- *V. unguiculata* Hb	This study
pESC-AN-gmaHb	D452-2 URA3, P_GAL1_-AN- *G. max* Hb	This study
pESC-AN-carLegHb	D452-2 URA3, P_GAL1_-AN- *C. arietinum* Hb	This study
pESC-AN-vraHb	D452-2 URA3, P_GAL1_-AN-*V. radiata* var. *radiata* Hb	This study
pESC-AN-tprHb	D452-2 URA3, P_GAL1_-AN-*T. pratense* Hb	This study
pESC-AN-vunHb	D452-2 URA3, P_GAL1_-AN-*V. unguiculata* Hb	This study
*K. phaffii* X-33	Wild type	Invitrogen
pPICzαA-MFα-gmaHb	X-33, P_AOX1_-MFα-*G. max* Hb	This study
pPICzαA-MFα-carHb	X-33, P_AOX1_-MFα-*C. arietinum* Hb	This study
pPICzαA-MFα-vraHb	X-33, P_AOX1_-MFα-*V. radiata* var. *radiata* Hb	This study
pPICzαA-MFα-tprHb	X-33, P_AOX1_-MFα-*T. pratense* Hb	This study
pPICzαA-MFα-vunHb	X-33, P_AOX1_-MFα-*V. unguiculata* Hb	This study
pPICzαA-HS-gmaHb	X-33, P_AOX1_-HS-*G. max* Hb	This study
pPICzαA-HS-carHb	X-33, P_AOX1_-HS-*C. arietinum* Hb	This study
pPICzαA-HS-vraHb	X-33, P_AOX1_-HS-*V. radiata* var. *radiata* Hb	This study
pPICzαA-HS-tprHb	X-33, P_AOX1_-HS-*T. pratense* Hb	This study
pPICzαA-HS-vunHb	X-33, P_AOX1_-HS-*V. unguiculata* Hb	This study
pPICzαA-AN-gmaHb	X-33, P_AOX1_-AN-*G. max* Hb	This study
pPICzαA-AN-carHb	X-33, P_AOX1_-AN-*C. arietinum* Hb	This study
pPICzαA-AN-vraHb	X-33, P_AOX1_-AN-*V. radiata* var. *radiata* Hb	This study
pPICzαA-AN-tprHb	X-33, P_AOX1_-AN-*T. pratense* Hb	This study
pPICzαA-AN-vunHb	X-33, P_AOX1_-AN-*V. unguiculata* Hb	This study

## Data Availability

The original contributions presented in the study are included in the article/Supplementary Material, further inquiries can be directed to the corresponding author.

## Supplementary Materials

The following supporting information can be downloaded at: https://www.mdpi.com/article/10.3390/foods14081422/s1. Figure S1. Construction of the expression plasmid for plant leghemoglobin secretory production in *S. cerevisiae* and *K. phaffii*; Figure S2. SDS-PAGE analysis of plant leghemoglobin concentrates after precipitation using ammonium sulfate; Figure S3. SDS-PAGE analysis of purified plant hemoglobins secreted by recombinant *S. cerevisiae* with different signal peptides; Figure S4. SDS-PAGE analysis of purified plant hemoglobins secreted by recombinant *K. phaffii* with different signal peptides.
