# Supplementary material for: Secretory Production of Plant Heme-Containing Globins by Recombinant Yeast via Precision Fermentation
Source: Foods. 2025 Apr 20;14(8):1422. doi: 10.3390/foods14081422 (PMC12026525; doi:10.3390/foods14081422)
Supplement: Supplementary file 1 [file foods-14-01422-s001.zip › foods-3568595-supplementary.pdf]

Supplementary Information

Secretory Production of Plant Heme-Containing Globins by Recombinant Yeast via Precision Fermentation

Ha-Neul Bae[1], Geun-Hyung Kim[1] and Seung-Oh Seo[1, 2*]

[1]*Department of Food Science and Biotechnology, Seoul National University of Science and Technology, Seoul 01811, Republic of Korea;*

[2]*Research Institute of Food and Biotechnology, Seoul National University of Science and Technology, Seoul 01811, Republic of Korea*

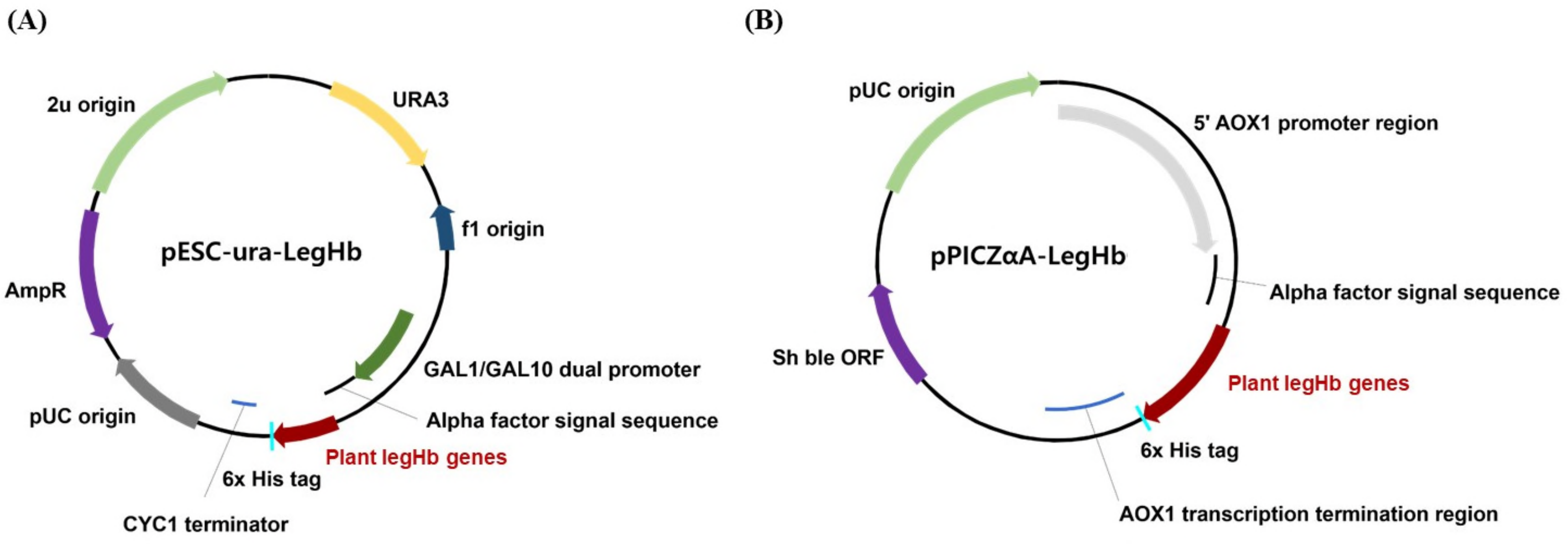

Figure S1. Construction of the expression plasmid for plant leghemoglobin secretory production in *S. cerevisiae* (A) and *K. phaffii* (B).

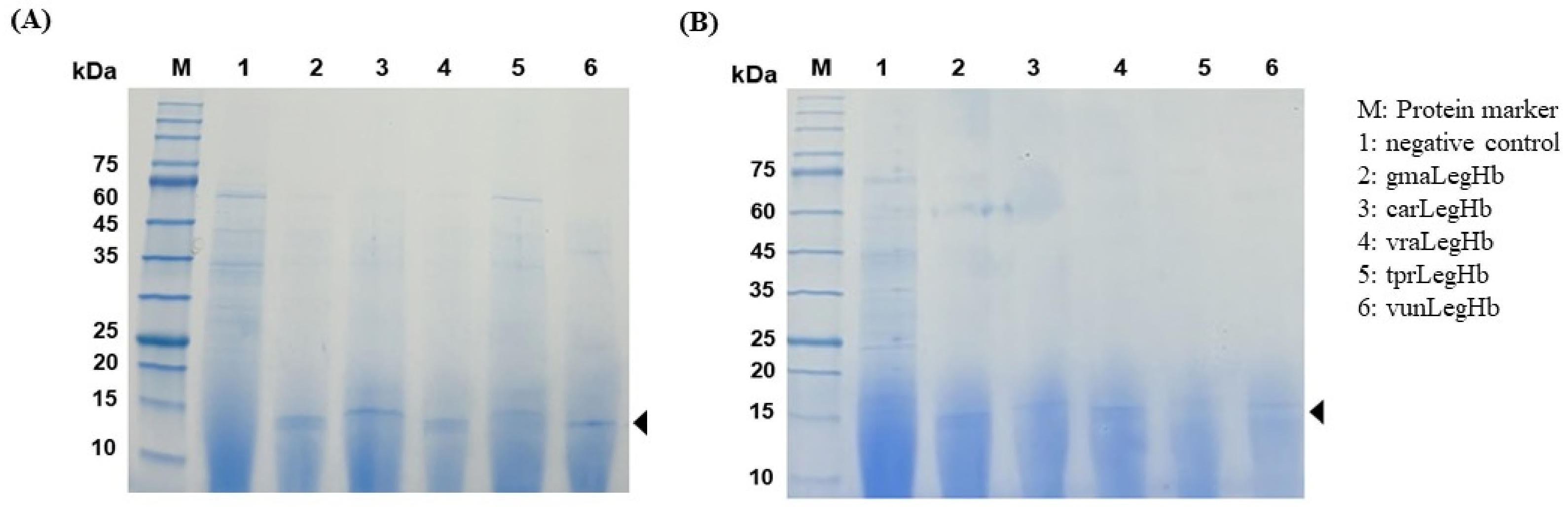

Figure S2. SDS-PAGE analysis of plant leghemoglobin concentrates after precipitation using ammonium sulfate.

(A) Leghemoglobin precipitates produced by the recombinant *S. cerevisiae* and (B) by the recombinant *K. phaffii* strains

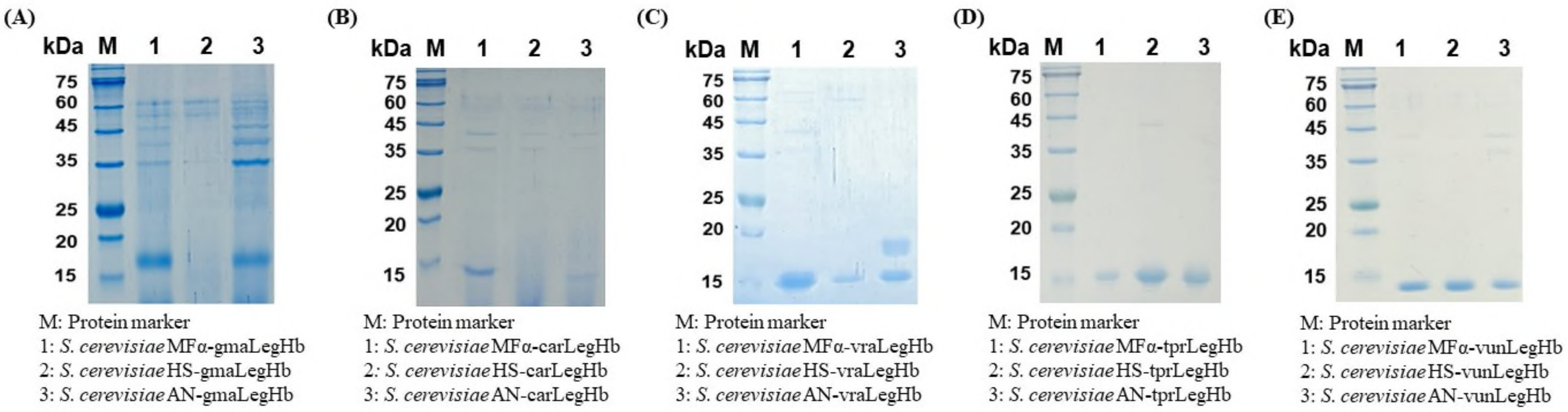

Figure S3. SDS-PAGE analysis of purified plant hemoglobins secreted by recombinant *S. cerevisiae* with different signal peptides.

(A) gmaLegHb, (B) carLegHb, (C) vraLegHb, (D) tprLegHb, (E) vunLegHb expression in *S. cerevisiae*

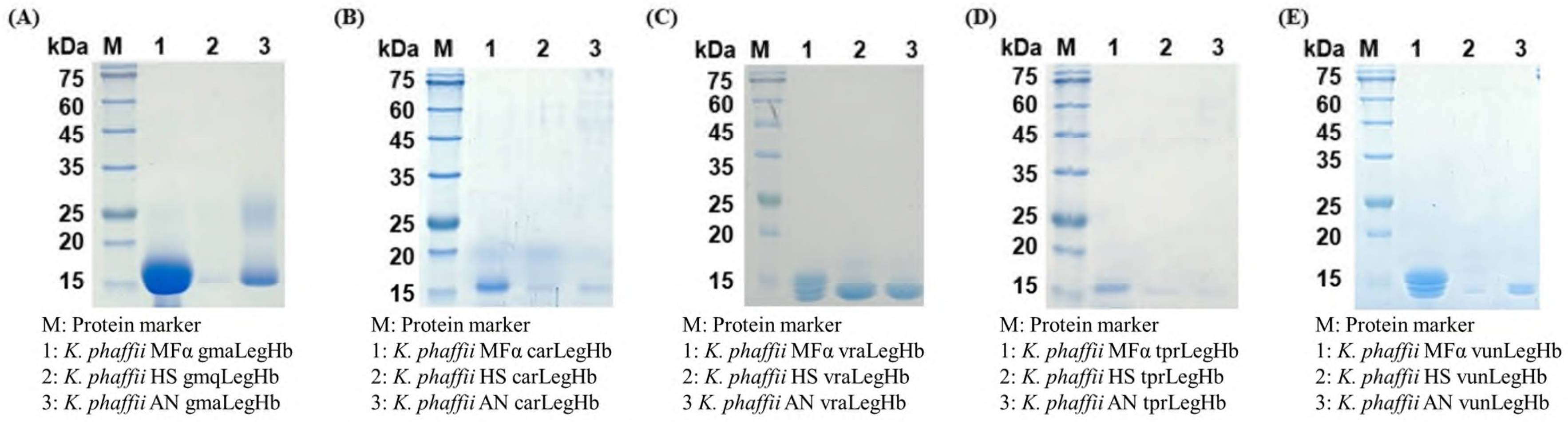

Figure S4. SDS-PAGE analysis of purified plant hemoglobins secreted by recombinant *K. phaffii* with different signal peptides.

(A) gmaLegHb, (B) carLegHb, (C) vraLegHb, (D) tprLegHb, (E) vunLegHb expression in *K. phaffii*
